# Hidden Costs of the COVID-19 Pandemic Response

**DOI:** 10.3390/ijerph20085476

**Published:** 2023-04-12

**Authors:** Sean G. Young

**Affiliations:** Peter O’Donnell Jr. School of Public Health, University of Texas Southwestern Medical Center, Dallas, TX 75235, USA; sean.young@utsouthwestern.edu

## 1. Introduction

“First, do no harm” [1]. This common rendition of the Hippocratic oath is familiar to most public health professionals, yet the practical application of this principle is often subjugated to the utilitarian principle of the most good for the most people [2]. If a minimal amount of avoidable harm to a few people will help avert a much larger amount of harm to many people, there is a utilitarian argument to be made for deliberately inflicting the lesser amount of harm. However, it should be noted that such a guiding principle is fraught with danger, which must be guarded against carefully, preferably through the use of rational and dispassionate assessments of costs and benefits, and a commitment to transparency and truth [2,3]. Such an approach can be expected to make relatively few mistakes (e.g., inflicting unnecessary or unjust costs) and, equally as important, should be able to recognize, admit, and correct mistakes when they occur. Alternative approaches to setting public health policy, such as top-down mandates and rejection of debate, lead to widespread mistrust and may end up causing more harm than the harm they purport to avoid. Some interventions ultimately make things worse, usually through indirect or hidden costs that are not adequately explored before or examined after implementation [4].

## 2. Case Study of Unintended Consequences

Consider the case of Cholera in Bangladesh. Cholera is endemic in Bangladesh, which essentially experiences a continuous stream of cases year round [5]. Most likely the very hearth of the cholera bacterium, Bangladesh has suffered greatly from this deadly disease for thousands of years [6]. Cholera is a waterborne diarrheal disease, spread in places such as Bangladesh through the use of infected surface waters and poor sanitary conditions, whereby wastes from sick individuals contaminate drinking water supplies. In the surge of scientific interventionism following WWII, millions of dollars and volunteer manhours were donated to rid the nation of this plague through the drilling of tubewells to provide fresh, bacteria-free water to the inhabitants. Major health education campaigns focused on cholera, and safe water practices in the 1970s helped convince large portions of the population to shift from their traditional use of surface waters to microbiologically safe tubewell water [7]. By the 1990s, nearly 100% of the population had access to tubewell water, and cholera incidence declined [8]. This appeared to be a clear win for public health intervention. However, in the early 1990s, the problem of naturally occurring arsenic in Bangladesh also came to light, and subsequent investigations demonstrated that 62 of 64 districts in the country were affected by arsenic contamination of groundwater. This massive multi-decade effort to move the population of Bangladesh from surface waters to groundwater from tubewells resulted in millions ingesting unsafe levels of arsenic in what has been called the largest mass poisoning in history [7,9].

It is not my intent to claim the public health interventions to combat cholera and other diarrheal diseases in Bangladesh were unethical or even necessarily a mistake. Hindsight is 20/20, and it is not clear that those involved knew about (or should have known about) the risk of arsenic poisoning or the extent to which Bangladesh was susceptible to it. Rather, I wish to use this case study merely to demonstrate that public health interventions can have unintended consequences, and these unintended consequences can be quite severe. Often, these costs are indirect or hidden, but that does not mean they cannot be identified and estimated. Even accepting a utilitarian ethic, the costs of interventions need to be weighed against their benefits if we want to guard against the possibility of the cure being worse than the disease.

## 3. Ethical Frameworks

Several public health ethical frameworks have been proposed to help guide interventions, often through posing a series of questions to be asked prior to intervention implementation [2]. For example, Kass (2001) suggested a 6-step framework with the following questions: 

(1) What are the public health goals of the proposed program? (2) How effective is the program in achieving its stated goals? (3) What are the known or potential burdens of the program? (4) Can burdens be minimized? Are there alternative approaches? (5) Is the program implemented fairly? (6) How can the benefits and burdens of a program be fairly balanced? [10]

A similar checklist of questions organized by key public health principles (Non-Maleficence, Beneficence, Health Maximization, Efficiency, Respect for Autonomy, Justice, and Proportionality) was proposed by Schröder-Bäck et al. (2014), including questions such as: 

Are especially children prevented from harm? Is the proposed intervention effective and evidence-based? Does the intervention refrain from employing coercion and manipulation? Is no one (including third parties) stigmatized, discriminated against, or excluded as a consequence of the proposed intervention? Does the intervention exacerbate social and health inequalities? Is the intervention the least infringing of possible alternatives? [11] 

These questions should be asked before, during, and after implementation, and the results of these ethical analyses should be made public and transparent to the maximum extent possible. Keep these questions in mind as we now turn to the topic of pandemic interventions.

## 4. COVID-19 Pandemic Response

Now, consider the varied COVID-19 pandemic responses across the world. Almost without exception, the worldwide public health response focused exclusively on reducing morbidity and mortality from COVID-19, with relatively little attention paid to the accompanying costs. Lockdowns, mask mandates, school closures, and other measures designed to reduce exposure to and transmission of the virus have numerous known externalities, but these have been largely downplayed, ignored, or even denied in the name of the greater good [3]. There is evidence that many COVID-19 studies overestimate benefits and underestimate the costs of lockdowns and other measures [12].

### 4.1. Benefits

Let us briefly explore some of the potential (and realized) benefits of the most common public health responses during the first year of the pandemic, prior to the widespread availability of vaccines and effective treatments. Lockdowns, including school closures, business closures, travel restrictions, curfews, and other rules enforcing social distancing, are intended to limit person-to-person contact and thereby limit the spread of the virus. Masks, both cloth/homemade and surgical, reduce the number of microorganisms through air filtration. In addition to these primary public health benefits, traffic reductions from reduced travel were recognized as conferring secondary benefits through reduced frequency of motor vehicle accidents and reduced air pollution [13,14].

The degree of benefit conferred by the above measures is an ongoing matter of dispute. Estimates rely largely on counterfactuals—estimating the number of deaths avoided in the “what if” scenario that lockdowns were not implemented [12]. These benefit estimates are often based on SIR compartmental epidemiological models, although simpler curve-fitting models have also been used, such as the IHME, which was particularly well publicized and influential in early pandemic planning [15,16]. These and other approaches to model and predict COVID-19 mortality under different policy scenarios show a wide range of predictions and substantial uncertainty, demonstrating the difficulty in modeling counterfactual scenarios [17,18,19,20].

Retrospective analyses and comparisons between regions and countries allow for a different approach to estimating the impact of various public health responses. Fukumoto et al. analyzed school closures in Japan in spring 2020 and found no evidence of reduced COVID-19 spread in the municipalities that closed schools [21]. Fuss et al. compared state and national lockdown policies and effectiveness at controlling the virus, including mortality, and found no significant impact of lockdowns [22]. Nanda et al. found poor evidence regarding the effectiveness of masks in protecting the wearer and no studies on the impact of viral spread [23]. While careful to point out that individual social distancing behaviors can be protective, Berry et al. found government-imposed restrictions and shelter-in-place orders were not effective and conveyed no detectable health benefits to the population [24]. There are, of course, also studies that support and promote these and other COVID-19 interventions, but there were and are sufficient concerns regarding effectiveness to suggest that alternatives should be carefully considered.

### 4.2. Costs

Setting aside, for the moment, the unresolved question of effectiveness of masks and lockdowns in conveying the intended benefits, let us examine some of the potential (and realized) costs.

Much has been said regarding the impact of masks, particularly on children; however, the evidence is mixed and of questionable quality. For example, many studies of speech delays and emotional recognition with masks rely on experimental designs using static images of masked faces, rather than in-person or video representations. Singh et al. found infants had no difficulty recognizing familiar words through opaque masks but did have trouble with clear masks, suggesting speech delays and other costs could be dependent on the surface material of the masks used [25]. Ruba and Pollack found some loss of emotional information transmission, comparable to that found when the subject wore sunglasses [26]. Schneider et al. found evidence of emotion misrecognition, and Gori et al. found that mask use interfered with the ability to accurately infer facial expressions at any age, but particularly in toddlers [27,28].

In addition to masks, lockdowns have measurable costs on children and youth, notably through the impact of school closures. Much could be said about the massive drop in math and reading scores across the United States, as shown in the 2022 National Assessment of Educational Progress, as well as the observed increases in racial disparities in test scores during the pandemic [29]. A study in the Netherlands, a so-called “best-case” scenario with a short lockdown, adequate and equitable school funding, and widespread broadband access, nevertheless, showed learning loss corresponding to the period of school closure, and also found that losses were more pronounced among students from less-educated homes, further exacerbating disparities [30]. Children are not the only ones to suffer from school closures. Medical students in Taiwan experienced significant deficits in clinical emergency training [31]. Lockdowns and school closures have also been linked to decreases in physical activity and substantial increases in screen time and negative emotions [32,33,34].

Perhaps the most obvious cost of lockdown orders was the astounding economic costs, due to business closures, changes in worker productivity, and changes in consumer behaviors [35]. From a purely economic standpoint, there is evidence that lockdowns can increase poverty and inequality [36]. Despite the objections of some to comparing economic costs with lives, economic costs have direct public health consequences that readily translate into costs in terms of lives. An individual who loses their livelihood will generally also lose health insurance benefits, have a greater risk of food insecurity, and be at greatly increased risk of mental health challenges, including drug and alcohol abuse and suicide, and this was directly observed during the COVID-19 pandemic [37,38]. As early in the pandemic as May 2020, Petterson et al. warned of increasing “deaths of despair”, such as suicides and drug overdoses, which are strongly correlated with increases in social isolation and economic stress, both hallmarks of the COVID-19 pandemic, which were magnified by the public health response [39]. Perhaps unsurprisingly, 2020 saw a record high number of drug overdose deaths in the United States [40]. In addition, reports of domestic abuse, food insecurity, and alcohol use and potential alcoholism all increased during the pandemic, often linked to economic stress and quarantines/lockdowns [41,42].

As early as February 2020, psychiatrists and researchers were warning of the impact of lockdowns and other responses on depression, anxiety, fear, panic attacks, suicidality, and other severe mental health problems, among both patients and health workers [43,44]. Studies around the world have found alarming rates of depression and stress on particular groups, such as caregivers of persons with dementia in Hong Kong (nearly two-thirds suffered from probable depression) [45], college students in Poland (over one-third reported poor mental health requiring treatment) [46], college students in China (nearly one-quarter reported mild-to-severe anxiety) [47], and young adults in Saudi Arabia (over 90% reported mild-to-severe symptoms of mental health disease) [48].

There are entire scientific journals dedicated to neglected diseases, but during the COVID-19 pandemic, it could be argued that nearly everything other than COVID-19 fell into that category. In some cases, the public health response, in concert with widespread public fear, led to disruptions even to emergency care. The CDC reported drops in emergency department visits during the 10 weeks following the declaration of the national emergency in the United States for numerous life-threatening conditions, including a drop of 20% or more for both heart attacks and strokes [49]. Lockdowns and the associated limitations placed on “elective care” in many hospitals and healthcare clinics led to demonstrable disruptions in preventative care. In 2020 alone, it is estimated that more than 9 million cancer screenings that would normally have taken place in the United States were missed, which could lead to delayed diagnoses at more advanced stages and higher cancer mortality burden in years to come [50]. In Lithuania, visits to physicians in 2020 dropped by 34%, visits to oncologists dropped by 20%, cancer screenings dropped by 28 to 43%, cancer diagnostic procedures dropped, and so on [51]. The questions of health equity and disproportionate risks and burdens have loomed large throughout the pandemic. Significant disruptions to childhood vaccination efforts were reported across the globe, including decreases in uptake for diphtheria tetanus pertussis, BCG, measles, and polio vaccines [52]. What is even worse is that the greatest drops were concentrated in countries that already experienced suboptimal vaccination rates, indicating spatial health inequalities were made more severe. In light of the ethical questions posed above, these unintended costs certainly reflect poorly on lockdowns as a public health intervention.

## 5. Conclusions

I have listed only a small sampling of the currently known hidden costs of the COVID-19 pandemic response. More could be said regarding food insecurity, smoking behavior, opioid use, as well as the incredibly controversial topics of COVID-19 vaccine efficacy and safety. I sincerely hope more is said about all of these costs, as well as the benefits. As these and other hidden or indirect costs have added up over time, it has become increasingly necessary to examine them more carefully to ensure a balanced and ethical evaluation of the COVID-19 pandemic response. This Special Issue is only one contribution to this effort, but much more is needed.

## Data Availability

No new data were created for this article.
